# A Global Review of Salt Marsh Restoration: Why Simpler, Context‐Specific Strategies Can Outperform Complex Approaches

**DOI:** 10.1111/gcb.70925

**Published:** 2026-05-21

**Authors:** Serena De Lauretis, Davide De Battisti, Ferrante Grasselli, Laura Airoldi

**Affiliations:** ^1^ Chioggia Hydrobiological Station “Umberto D'Ancona”, Department of Biology University of Padova, Uo CoNISMa Chioggia Italy; ^2^ NBFC, National Biodiversity Future Center Palermo Italy

**Keywords:** coastal ecosystems, context dependency, ecosystem recovery, meta‐analysis, reference conditions, restoration outcomes, salt marsh restoration

## Abstract

Improving our understanding of the drivers of restoration success is a global priority, as effective restoration efforts are essential for biodiversity conservation, climate resilience and regenerative development. We present a global meta‐analysis of active salt marsh restoration to evaluate how different restoration approaches approximate natural reference conditions across physical, biotic, and functional attributes, how factors such as project age, project timing, spatial extent, and latitude influence outcomes, and whether combining interventions yields synergistic benefits over single methods. Most approaches, including tidal restoration, sediment addition, and fertilisation, approximated reference conditions on average, but outcomes varied widely among studies, with attributes often falling short of or exceeding those of natural sites. The diversity of metrics used to assess restoration outcomes further complicates the interpretation of this variability. Deviations from reference conditions did not diminish with increasing project age, spatial extent or timing, highlighting the strong context dependency of restoration outcomes and the need for improved evaluation frameworks. Contrary to prevailing assumptions, combining multiple restoration interventions did not consistently enhance restoration success and was also associated with lower similarity to reference conditions and greater variability in outcomes. However, this pattern appears to be driven by the performance of specific intervention combinations rather than reflecting a general disadvantage of multi‐intervention approaches. These findings suggest that combinatorial restoration strategies are heterogeneous in effectiveness and may be less predictable across contexts than simpler interventions, emphasising the need for careful restoration planning and indicating that context‐specific strategies may provide more scalable solutions under global change.

## Graphical abstract

Salt marshes are vital coastal ecosystems, yet restoration outcomes remain highly variable and difficult to predict. We present the first global meta‐analysis comparing active salt marsh restoration interventions across physical, biotic and functional attributes. Most single interventions, on average, approximated natural reference conditions, but outcomes varied widely among studies. In contrast, combining multiple interventions often reduced restoration success (i.e., similarity to reference conditions) and increased variability. These findings highlight persistent uncertainty in restoration practice and underscore the need for context‐dependent strategies, cautioning against increasingly complex restoration designs without clear evidence of their effectiveness.

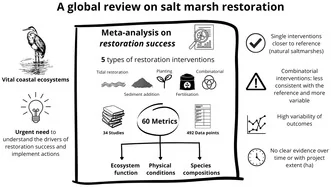

## 1 Introduction

Restoration is increasingly used to counter global environmental degradation, driven by the recognition of the ecosystem services provided by natural and novel ecosystems (Benayas et al. 2009; Hobbs et al. 2009). Initiatives like the UN Decade on Ecosystem Restoration and the EU Nature Restoration Law promote approaches such as building with nature and nature‐based solutions, which combine restoration with structural elements to maximise ecological and social co‐benefits through context‐specific design (Airoldi et al. 2021; Perricone et al. 2023). Despite these growing efforts, there are still persistent limitations in restoration science and practice, and improving our understanding of the drivers of restoration success is a global priority (Airoldi et al. 2025; Sievers et al. 2024). Although ecological restoration faces inherently complex and site‐specific challenges, ranging from hydrology and soil dynamics to biodiversity goals, implementation often defaults to a narrow set of standardised techniques deployed in isolation. This ‘one‐size‐fits‐all’ tendency can limit success, as mounting evidence shows that restoration success depends on tailoring interventions to local ecological drivers (Strain et al. 2020). There has been an emerging proposition that restoration outcomes could be improved by combining complementary approaches (Rillig et al. 2024), as this could produce synergistic effects, with each component compensating for the limitations of others. For example, combining sediment addition with planting may simultaneously restore elevation and accelerate vegetation recovery, while hybrid approaches that merge physical and biological interventions are thought to increase system resilience (Sutton‐Grier et al. 2015). These combinatorial strategies are conceptually appealing because they promise to tackle multiple constraints at once and achieve more restoration outcomes that are better tailored to local drivers (Rillig et al. 2024). However, despite their growing popularity in theory and practice, empirical evidence supporting the consistent effectiveness of such approaches remains limited and fragmented. Furthermore, most evaluations focus narrowly on structural attributes (e.g., vegetation cover or habitat area), yet success across multiple outcome dimensions, such as habitat structure, biodiversity and ecosystem functioning is essential for assessing whether restored systems are delivering intended benefits. Significant uncertainties also remain about where restoration should be prioritised, how long recovery takes, and what scale of intervention is needed to achieve lasting ecosystem resilience, all of which underscore the importance of context‐specific planning.

Salt marshes are coastal ecosystems that provide numerous benefits to human populations, from coastal protection to carbon sequestration and water quality (Barbier et al. 2011; Howard et al. 2017; Koch et al. 2009). Their ecosystem services value ranks among the highest in nature (Costanza et al. 1997), yet human activities and climate change have severely degraded salt marshes (Gedan et al. 2009; Strain et al. 2017) with global losses estimated at 46%–50% (Davidson 2014). Growing recognition of their ecological and economic importance, including their role in cost‐effective nature‐based solutions for erosion and flooding, is driving major restoration investments (Adam 2019; Vozzo et al. 2024; Zedler and Kercher 2005).

Salt marsh restoration approaches are diverse in both objectives and methodologies and have been refined over several decades of practice to suit a wide range of environmental, economic and cultural contexts (Bekkby et al. 2020; Zhao et al. 2016). These approaches encompass a variety of active biotic and abiotic manipulations, including planting of vegetation (Broome et al. 1988; Castillo and Figueroa 2009; Zengel et al. 2021), the use of structures to enhance plant recruitment and transplant survival (Temmink et al. 2020), the addition of dredged sediments to restore marsh elevation (Scarton et al. 2013; VanZomeren et al. 2018), the de‐embanking of old artificial levees to re‐establish natural tidal flow (Burdick et al. 1996; Wolters et al. 2005), and the application of fertilisers to stimulate vegetation growth and boost marsh recovery (Boyer and Zedler 1999; Vieillard and Fulweiler 2012). The outcomes are often variable, but what shapes this variability is often unclear (Airoldi et al. 2025; Kutcher and Raposa 2023; Wolters et al. 2008). In recent years, hybrid approaches such as living shorelines (Davis et al. 2015), which integrate screens, fences, and natural or artificial reefs to protect salt marshes from waves and predators, and multi‐layered approaches combining sediment addition with vegetation planting (Rozas and Minello 2001), have gained popularity due to their perceived greater success. This array of approaches makes salt marshes an ideal system for exploring the multifaceted effectiveness of active restoration, including direct comparison between individual and combinatorial approaches, and for identifying the factors that can enhance restoration success.

We conducted a comprehensive global meta‐analysis of active salt marsh restoration practices aiming to: (1) evaluate the overall success of salt marsh restoration across a range of physical, biotic and functional attributes; (2) assess how success is influenced by factors such as project timing (year when the restoration was initiated, which can be related to technological or methodological advancements), age (i.e., time elapsed since the intervention, which measures the time for ecosystems to recover), spatial extent (area of habitat recovered), or latitudinal context (which reflects broad environmental gradients); (3) determine whether some intervention approaches consistently outperform others; and (4) assess whether combining multiple restoration approaches (e.g., sediment addition and planting) leads to synergistic gains in restoration outcomes compared to applying each approach in isolation. For the purpose of this review, we define restoration success as the degree of alignment between actively restored salt marshes and natural reference systems, measured across physical, biotic or functional attributes. We recognise, however, that similarity to reference conditions represents only one dimension of restoration outcomes, and that under ongoing environmental change, functionally resilient but compositionally novel states may also represent valid restoration trajectories. We also tried to include the complementary option, that is the degree of divergence from degraded unrestored conditions, but we could not find enough papers to run a robust meta‐analysis. We also evaluated the variability of restoration outcomes as a measure of consistency, as consistent outcomes build confidence in the ability to reliably predict and replicate results at new sites, thereby supporting broader implementation. Here we deliberately focus on active restoration (which relies on human innovation and technology) rather than passive restoration (focusing mostly on controlling human disturbances). This focus reflects the need for greater confidence and investment in active restoration, as such interventions are essential to address the magnitude and pace of current biodiversity decline. We acknowledge, however, that passive restoration is often a prerequisite for successful active restoration; without addressing the underlying causes of degradation, active interventions may fail or achieve only limited outcomes (Liu et al. 2021).

This synthesis provides critical evidence to guide more successful and resilient restoration planning in the face of accelerating climate and anthropogenic pressures on coastal ecosystems.

## 2 Materials and Methods

### 2.1 Literature Search

In July 2022, we searched the literature using Scopus and Web of Science for manipulative field studies that tested the effects of salt marsh restoration methods. The search string was: “restor* OR recover OR enhanc* OR requalification OR rehab* OR reclaim OR remediation OR manipulat* OR transplant* OR ‘soil amelioration’ OR ‘soil amendment’ OR protect* OR ‘soil enhancement’ OR fertiliz* AND cordgrass OR salt marsh* OR ‘salt marsh*’ OR marsh* OR halophyt* OR (wetland* AND (estuarine OR tidal OR coastal)) OR ((vegetation OR trait*) AND (emergent)) AND (techniqu* OR approach* OR measur* OR solution* OR material* OR intervention* OR (substrat* OR anchorag*) AND artificial) OR ‘establishment structur*’ OR application* AND grow* OR recruitment OR settlement OR survival OR success OR density OR cover* OR yield* OR efficac*”.

We searched within article titles, abstract and keywords, including only peer‐reviewed papers. A total of 6980 publications were identified, reduced to 431 potential publications after duplicate removal and screening of the titles. The remaining 431 papers were screened in a two‐step process: first, abstracts were assessed for reporting the outcomes of active in situ salt marsh restoration, which led to 209 papers; second, two investigators independently reviewed each study's full text to promote unbiased inclusion of papers that assessed the outcomes of the restoration relative to at least one unrestored ‘degraded’ or reference ‘natural’ salt marsh. This process led to a final list of 63 retained papers (the articles included in this review are listed in the ‘Data Sources’ section), including two papers retrieved through ‘snowballing’ (Wohlin et al. 2022). The literature review was conducted according to the Preferred Reporting Items for Systematic Reviews and Meta‐Analyses (PRISMA) guidelines (Stovold et al. 2014). A PRISMA flow diagram showing the literature selection procedure is provided in Figure S1.

### 2.2 Data Extraction

From each of the 63 eligible papers (identified by a unique Study ID), we systematically extracted key contextual and methodological information to enable robust comparisons across restoration efforts. Specifically, we recorded: the type of vegetation restored, geographic location, tidal regime, type of intervention (i.e., vegetation planting, sediment addition, chemical fertilisation, tidal restoration, use of fences or reefs, use of enhancing structures), whether the intervention was single or combinatorial, the age (in years) at the time of monitoring, project timing (i.e., year when the restoration was initiated), spatial extent (area of habitat recovered) and latitude of each intervention. In line with the standardised framework proposed by Bayraktarov et al. (2020), we also documented which outcome metrics were measured to estimate restoration success (Table 1). We acknowledge that alternative classifications are possible and that these categories are not strictly mutually exclusive; our approach represents a pragmatic, literature‐based framework designed to ensure consistency and reproducibility in synthesis rather than to provide a definitive ecological partitioning. From a subset of 34 papers (Table S1), it was possible to further extract the necessary quantitative information—sample size, mean and standard deviation (SD)—to perform the subsequent meta‐analyses. Some studies compared more than one restoration approach or measured several outcome metrics: these were recorded as individual data points in the spreadsheet, each linked by an experimental identification number (Exp ID) nested within the corresponding Study ID. When means and associated variability were not reported in the text or tables, we extracted those from plots using GetData Graph Digitizer version 2.26. In addition, all standard errors (SE) were converted to SD. When standard deviations (or equivalent measures) were missing from publications, we requested the data from the authors. If this information remained unavailable, we imputed SD values following the method described by Furukawa et al. (2006), which estimates SD from the relationship between mean, sample size and SD derived from the available dataset. This imputation was applied to 20 data points from 5 different studies.

**TABLE 1:** Quantitative restoration outcome metrics extracted from the papers included in the meta‐analysis (Table S1).

Attribute and sub‐attributes
**Ecosystem function**
*Blue carbon*
CH₄ fluxes (μmol m^−2^ h^−1^)
*Growth and productivity*
Vegetation cover (% per unit area), stem height (cm), stem density (no. m^−2^), root density (mg cm^−3^), biomass (g‐Kg m^−2^), leaf area (cm^2^), gross oxygen production (GOP, mmol O_2_ m^−2^ s^−1^), net annual carbon capture (NACC, kg m^−2^), net annual primary production (NPP, kg m^−2^), chlorophyll (μg g^−1^)
*Habitat function*
Invertebrate biomass (g or kg), invertebrate density (no. ha^−1^ or no. m^−2^) (molluscs, crustaceans, polychaetes, insects), density of epifauna (no. m^−2^), density of infauna (no. m^−2^), fish biomass (g or kg), fish density (no. ha^−1^ or no. m^−2^), vegetation diversity (Simpson's index), trophic diversity (SEAc, isotopic niche area, ‰^2^), trophic redundancy (NND, nearest neighbour distance, ‰)
*Nutrient cycling*
Leaf nutrients (C, N, and C:N ratio, %), decomposition rate (K), denitrification (μmol N_2_O g^−1^ dry soil h^−1^), N fixation rate (μmol N m^−2^ d^−1^), short‐term C accumulation rate (SCAR, cm yr.^−1^), microbial biomass carbon (MBC, g kg^−1^), microbial biomass nitrogen (MBN, mg kg^−1^)
*Sediment trapping*
Accretion rate (mm yr.^−1^)
**Physical conditions**
*Physical attributes*
Salt marsh elevation (cm or m), mean grain size (μm), mean high and low water (cm), rugosity (m, SD of elevation), relative topographic position, time flooded (%), clay (%), silt (%), sand (%)
*Physico‐chemical variables (soil)*
Organic matter (OM, %—mg g^−1^), total C (g kg^−1^), water content (%), pH, bulk density (g cm^−3^), porewater salinity (ppt), C:N ratio, redox potential (mV), mineral matter (mg g^−1^), soil stability (psi)
*Physico‐chemical variables (water)*
Total Kjeldahl nitrogen (TKN, mg L^−1^), salinity (ppt), temperature (°C), depth (cm), dissolved oxygen (DO, ppm)
*Rate of sedimentation*
Accretion rate (cm yr.^−1^), sediment deposition (kg m^−2^ yr.^−1^)
**Species composition**
*Growth and productivity of species*
Cover of more than one halophyte species (%)
*Species composition (non‐target fauna)*
Density of fishes, molluscs, crustaceans (no. m^−2^), biomass of fishes, molluscs, crustaceans (g)
*Species composition (non‐target flora)*
Cover (%)
*Species makeup, diversity and distribution*
Alpha diversity, beta diversity, gamma diversity, Shannon diversity, species richness

*Note:* Metrics are hierarchically categorised into ecosystem function, physical conditions, and species composition, and their related sub‐attributes, following the classification of Bayraktarov et al. (2020). Details of the metrics can be found in their Supporting Information (10.3389/fmars.2020.00484/full#supplementary‐material).

At the end of this procedure, we obtained 492 individual data points covering 60 different outcome metrics across three attribute categories (ecosystem function, physical conditions and species composition) and types of single or combinatorial intervention (see standard in Results). However, reef creation/fencing and enhancing structures lacked sufficient replication and were excluded from further analyses.

For this study, combinatorial approaches refer to interventions that integrate two or three of the primary restoration approaches (e.g., sediment addition plus planting) (Figure S2). All 34 studies included in the meta‐analysis used “natural” salt marshes as reference sites. Studies that used impacted or degraded systems as references were excluded due to insufficient replication, which limited the ability to draw robust comparisons.

### 2.3 Meta‐Analysis

All analyses were performed in R version 4.3.2 (R Core Team 2024) and the code and data supporting this study are publicly available in Zenodo (see Data Availability Statement). We fitted different multi‐level models to test the effects of different moderators on restoration success. The moderators were: type of intervention (tidal restoration, fertilisation, planting, sediment addition and combinatorial) in interaction with outcome attributes (ecosystem function, physical conditions and species composition). We compared restored and reference sites using the natural logarithm of the response ratio (lnRR) as the effect size, which is the proportional change in yield between restored and natural salt marshes (Hedges et al. 1999). To quantify the effect of different restoration interventions on the variability of restoration outcomes we used the natural logarithm of the coefficient of variation (lnCVR) (Atkinson et al. 2022; Nakagawa et al. 2023). Both lnRR and lnCVR are dimensionless metrics and reflect the proportional difference between the treatment (i.e., the restoration intervention) and the control (reference natural salt marsh), independently of the units or absolute scale of the measured variables.

Where more than one restored salt marsh was compared to the same reference salt marsh, we used a modified sample size (*n*) in the effect size calculations to account for non‐independence (Higgins et al. 2019).

For the analysis of restoration success across interventions, we fitted a multilevel model with lnRR as the response variable and the interaction between intervention type and outcome attribute as fixed moderators. To evaluate variability in restoration outcomes, we fitted a separate multilevel model using the natural logarithm of the coefficient of variation ratio (lnCVR) as the response variable, with intervention type included as a fixed moderator.

We did not include age as a categorical fixed or random factor in the multi‐level models because data were strongly unbalanced across age classes, with limited replication of older restorations and sparse combinations across different intervention types, which would have reduced statistical power and potentially biased model estimates. Restoration age, as well as other contextual drivers (age, project timing, spatial extent and latitude) were therefore analysed as continuous moderators in separate meta‐regressions to explicitly account for their effects on lnRR. Each model included a random factor (Exp_ID) nested in Study_ID to account for non‐independence of multiple data points extracted from the same study (Noble et al. 2017). Multi‐level models for both lnRR and lnCVR were fitted using the *rma.mv* function from the *metafor* package (Viechtbauer 2010) and were run without an intercept to test whether there were significant differences between restored and reference salt marshes at each level of the factor considered. We used forest (*ggplot2* package) (Wickham 2016) and orchard plots (*OrchaRd* package) (Nakagawa et al. 2023) to visualise the effect sizes across different moderators, displaying 95% confidence intervals (CIs) and 95% prediction intervals (PIs). CIs relate to the most likely location of the cross‐study average effect, while PIs, also known as credibility intervals, incorporate variability and provide a range within which an individual observation is expected to fall (Nakagawa et al. 2023).

To assess whether the pooled effect attributed to combinatorial interventions was disproportionately influenced by restoration subtypes, we conducted additional leave‐one‐subtype‐out sensitivity analyses within the combinatorial intervention category. Specifically, each combinatorial subtype was sequentially excluded while retaining all remaining intervention categories in the dataset, and the multilevel meta‐analytic models were re‐fitted for both restoration effectiveness (lnRR) and variability (lnCVR).

## 3 Results

### 3.1 Qualitative Synthesis

**FIGURE 1:**
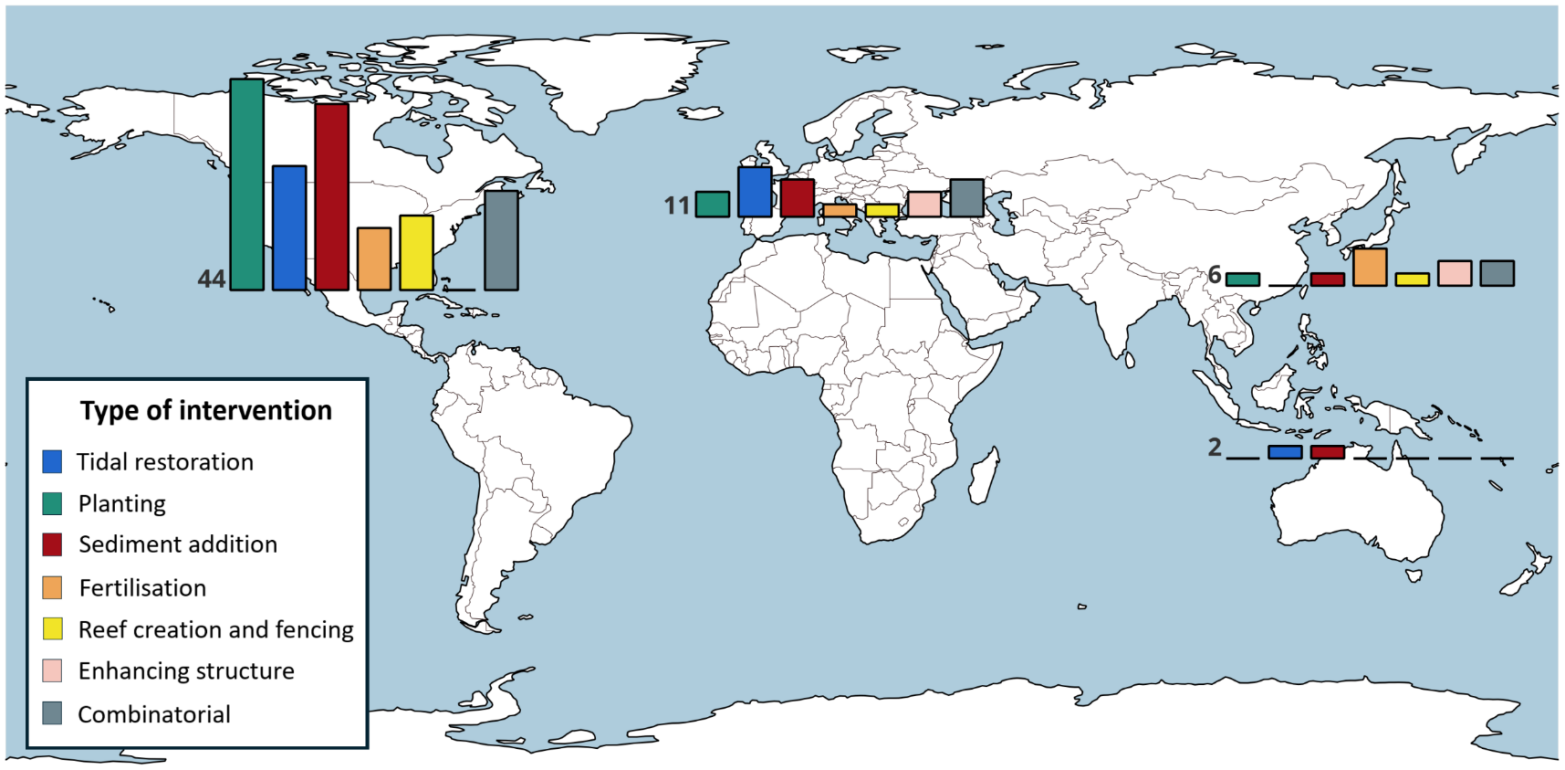
Thematic world map showing the continental distribution of the types of restoration interventions across the 63 eligible studies. The total number of studies per continent is reported to the left of each histogram. Map lines delineate study areas and do not necessarily depict accepted national boundaries.

**FIGURE 2:**
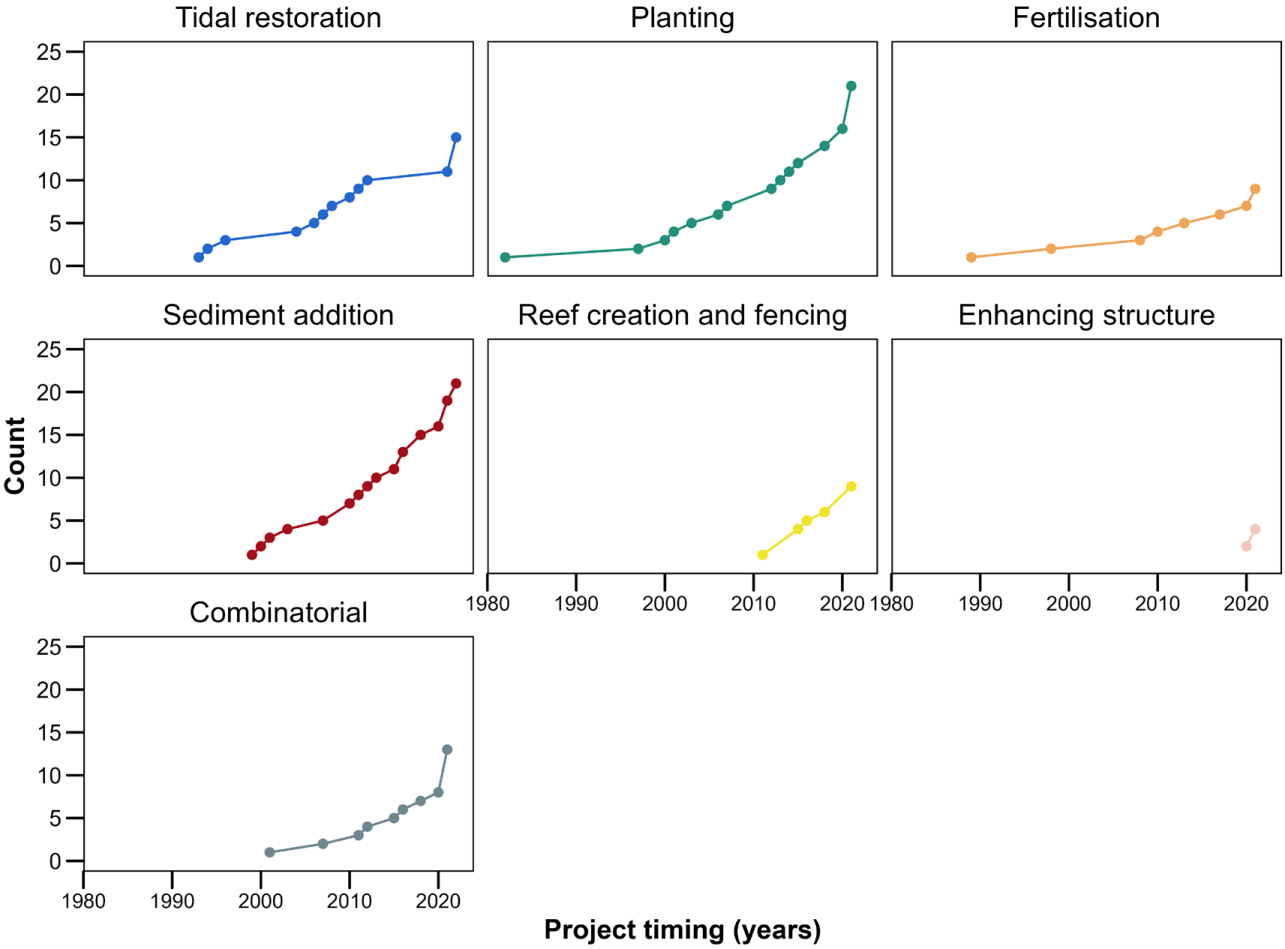
Cumulative number of studies for each type of restoration intervention over time, based on the year in which restoration was initiated (Project timing).

Most studies were performed in the Northern Hemisphere (97%), primarily in North America (USA and Canada, 69%), followed by Europe (18%), Asia (10%) and Oceania (3%) (Figure 1). No studies from South America or Africa were included because none meeting our inclusion criteria were identified. Planting, sediment addition, and tidal restoration were the most used restoration interventions, particularly in North America and Europe (Figure 1). In contrast, fertilisation and reef creation/fencing were uncommon in these regions but were the most frequently used approaches in Asia. Combinatorial interventions were used in all continents, except for Oceania where only sediment addition and tidal restoration were employed. The combinations of interventions included: sediment addition plus planting, sediment addition plus planting plus reef creation and fencing, sediment addition plus reef creation and fencing, and sediment addition plus tidal restoration (Figure S2). The spatial extent (i.e., the area of habitat recovered) of the restoration interventions ranged from 0.003 to 148 ha, with most studies below 20 ha in extent (75%). Age ranged from 6 months to 33 years, with most studies below 10 years of age (71%). Project timing ranged from 1982 to 2022, with an increase in the number of projects over time (Figure 2). Planting and fertilisation were the first type of restoration interventions reported (starting in the 1980's), followed by tidal restoration, sediment addition, and reef creation and fencing. Enhancing structures were the most recent restoration intervention, being reported only since 2020 (Figure 2).

### 3.2 Quantitative Meta‐Analysis

**FIGURE 3:**
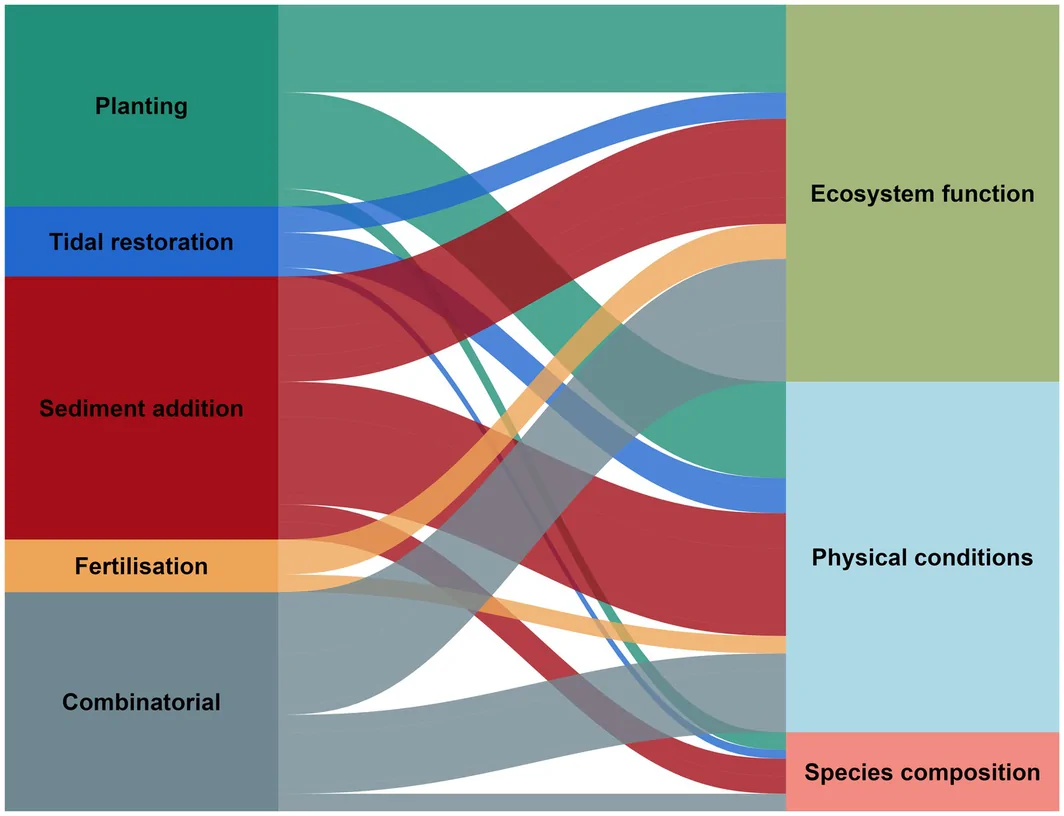
Sankey diagram showing the distribution of studies across restoration interventions and attribute categories. Line thickness indicates the number of studies connecting each restoration intervention (left) to a specific attribute category (right).

Among the 492 data points extracted from the 34 eligible papers, 65 (13%) pertained to species composition, 200 (41%) to physical conditions, and 226 (46%) to ecosystem function. These data were unevenly distributed among the different types of restoration interventions, with 59 data points (11%) for tidal restoration, 16 (3%) for fertilisation, 65 (13%) for planting, 149 (30%) for sediment addition and 203 (43%) for combinatorial approaches (Figure 3).

**FIGURE 4:**
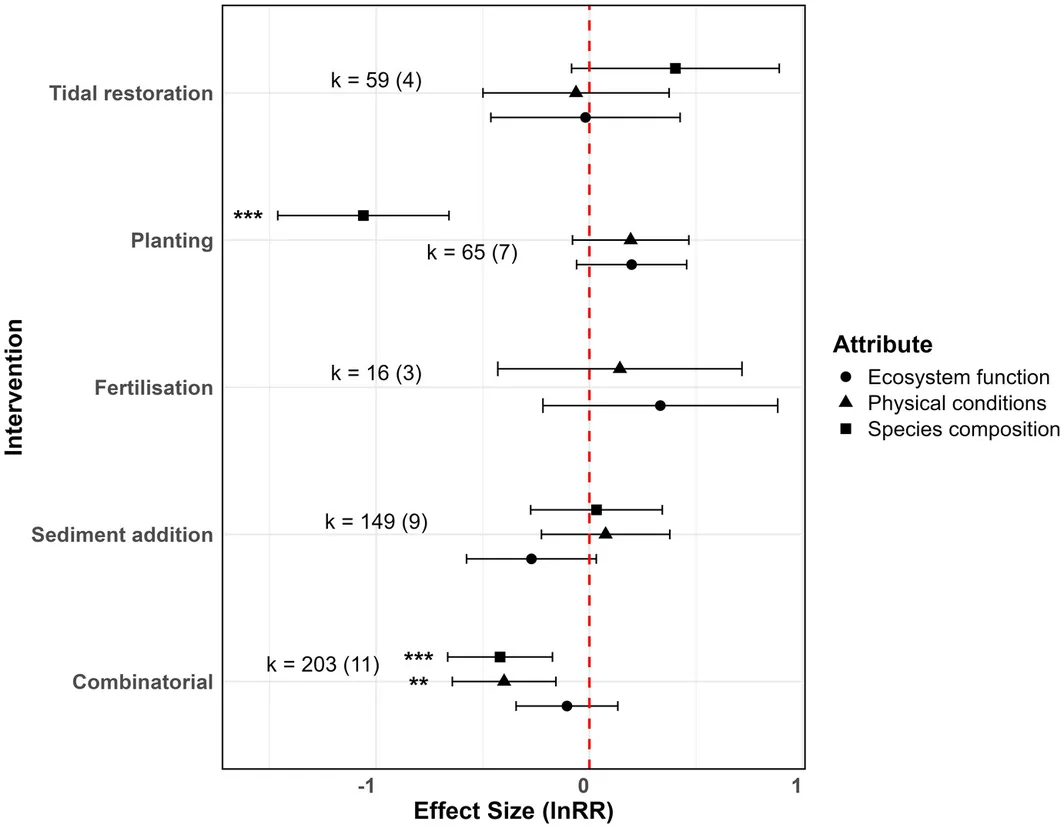
Effect sizes (lnRR ± 95% CI) of the five types of restoration interventions grouped by attribute category. Values near 0 indicate conditions comparable to the reference, natural salt marshes. “*k*” denotes the number of effect sizes (data points) per intervention, with the numbers of studies in parentheses. Asterisks indicate significant differences from the reference natural salt marshes (red dashed line at 0, representing the natural reference) at *p* < 0.01 (**), and *p* < 0.001 (***).

The success of the different restoration approaches depended on the outcome metrics assessed (interaction results, Table S2). When applied individually, most interventions (fertilisation, sediment addition, and tidal restoration) on average reasonably matched the reference conditions of natural salt marshes across most attributes (Figure 4). Furthermore, no significant differences in ecosystem function were observed between restored and reference sites across the five restoration interventions (Figure 4; Table S1). Planting was the only individual intervention that failed to replicate reference conditions in terms of species composition (lnRR = −1.06, *p* < 0.001). Compared to individual interventions, combinatorial ones were generally less successful in restoring both biotic and physical attributes (lnRR = −0.40, *p* = 0.001; lnRR = −0.42, *p* < 0.001, respectively) (Figure 4) (Table S2). However, functional attributes at sites restored with combinatorial interventions did not significantly differ from those of reference conditions (lnRR = −0.1, *p* = 0.4).

**FIGURE 5:**
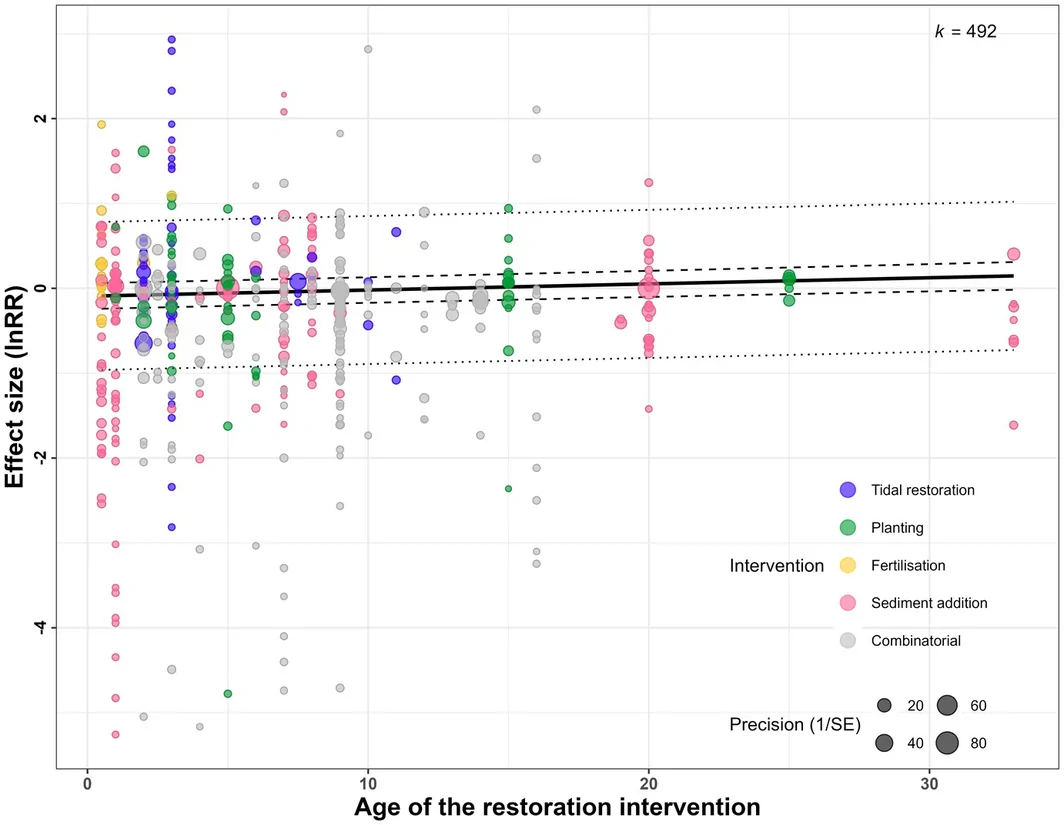
Relationship between age (in years) and restoration success (492 effect sizes). The solid line represents the fitted trend, with 95% confidence intervals shown as dashed lines and prediction intervals as dotted lines. Individual data points are scaled by precision (1/SE) and colour‐coded according to the type of intervention.

The meta‐regression analyses showed that restoration success did not significantly improve with age (lnRR = 0.02, *p* = 0.6) (Figure 5; Table S3). Rather, combinatorial approaches showed a significant negative main effect (lnRR = −1, *p* < 0.001), indicating that their similarity to reference conditions decreased as time progressed (Figure S3; Table S3).

**FIGURE 6:**
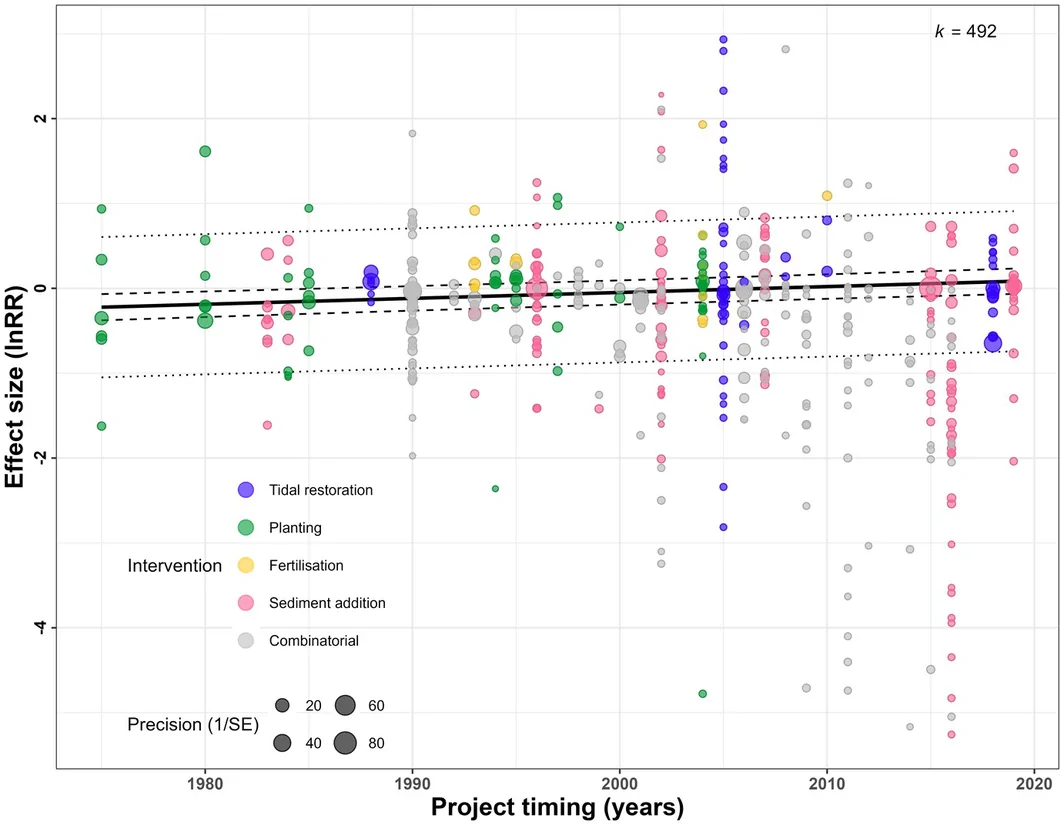
Regression line from meta‐regressions of all 492 effect sizes against project timing. The solid line shows the fitted trend, with 95% confidence intervals indicated by dashed lines and prediction intervals by dotted lines. Individual data points are scaled by precision (1/SE) and colour‐coded by type of intervention.

Similarly, restoration success did not improve with project timing, despite accumulated technological and methodological experience in the field (*p* = 0.66) (Figure 6; Table S4). In contrast, combinatorial approaches appeared to have become less successful over time (lnRR = −0.095, *p* < 0.001) (Figure S4, Table S4), gradually diverging from the alignment with reference conditions achieved by the earliest combinatorial ones in the 1990s. Additionally, the extent of the intervention did not improve restoration success at matching reference conditions (Table S5). Latitude affected restoration outcomes only in the tidal restoration (lnRR = −0.2, *p* = 0.04) (Table S6), with outcomes becoming more variable and deviating negatively from reference conditions at higher latitudes (Figure S5).

**FIGURE 7:**
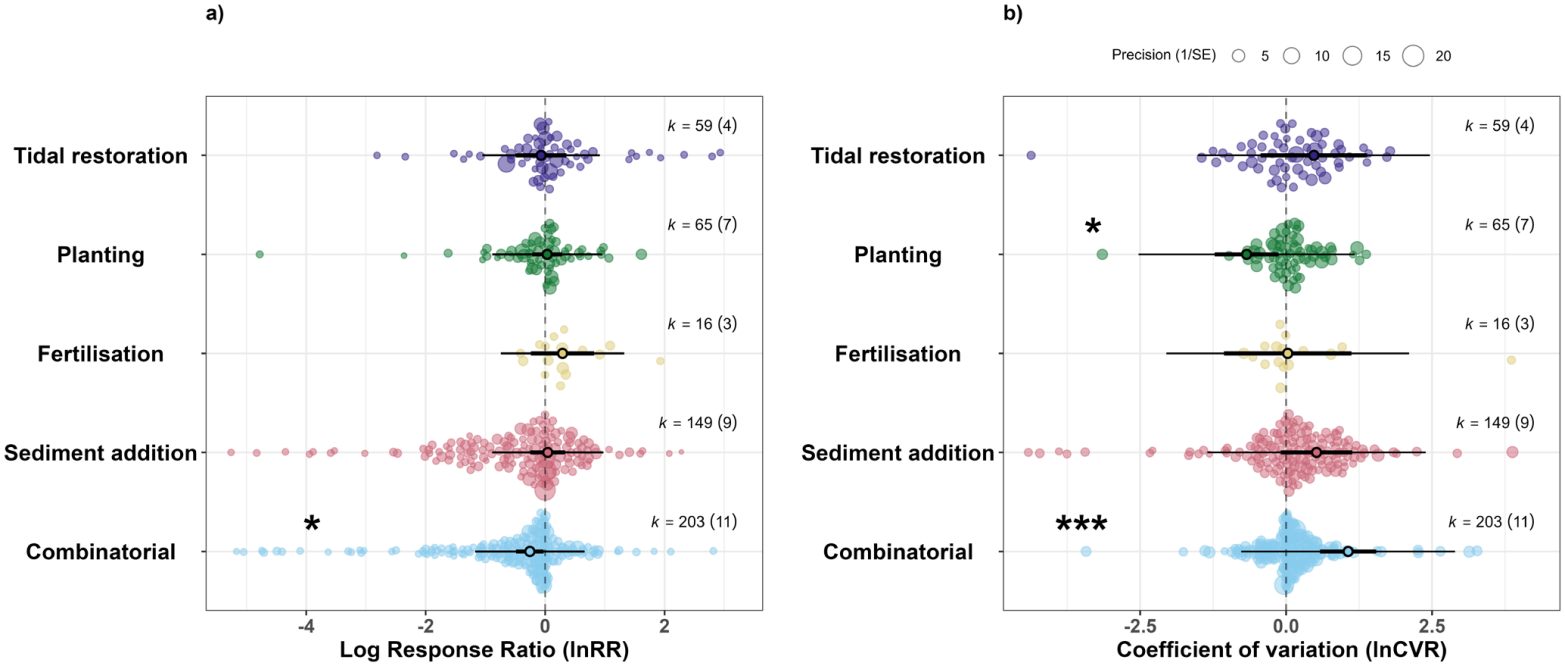
(a, b) Meta‐analysis comparing the moderating effects of the five restoration interventions: (a) means (LnRR) and (b) variability (LnCVR). Central points show model‐estimated means, with thick bars for 95% CIs and thin bars for prediction intervals. Values near 0 indicate conditions comparable to reference, natural salt marshes. For lnCVR, positive values correspond to increased variability and vice versa for negative values. “*k*” denotes the number of effect sizes (data points) per type of intervention with the numbers of studies in parentheses. Individual data points are shown, highlighting the high variability in effect sizes. Asterisks indicate significant differences from the reference natural salt marshes at *p* < 0.05 (*), and *p* < 0.001 (***).

Overall, the restoration success, measured as the degree to which restored salt marsh conditions matched reference marshes, was not achieved by combinatorial approaches only (lnRR = −0.26, *p* = 0.03) (Figure 7a; Table S7a). Furthermore, these approaches led to a substantial increase in post‐restoration variability, as indicated by a positive lnCVR value (1.06, *p* < 0.0001) (Figure 7b; Table S7b). In contrast, planting showed a significant reduction in variability after restoration compared to reference conditions (lnCVR = −0.68, *p* = 0.02) (Figure 7b; Table S7b). Overall, the analyses revealed substantial residual heterogeneity among studies, with large variability in effect sizes not fully explained by the moderators (type of intervention and attributes; Tables S8–S11).

Sensitivity analyses indicated that the pooled estimates for combinatorial interventions were influenced unevenly by individual subtypes. For lnRR, excluding the most abundant and complex subtype (sediment addition plus planting plus reef creation and fencing) shifted the pooled estimate towards the reference condition, reducing the magnitude of the overall effect relative to the full model. Excluding other combinatorial subtypes produced comparatively smaller changes in the pooled estimate (Table S12; Figure S6). For lnCVR, excluding the same complex subtype was associated with a noticeable reduction in outcome variability relative to the full model, whereas excluding the other subtypes resulted in little change (Table S13; Figure S7).

## 4 Discussion

Our qualitative synthesis and quantitative meta‐analysis provide critical insights into the effectiveness of active salt marsh restoration interventions and the methods used to evaluate them. While most interventions approximated, on average, reference conditions, individual studies exhibited considerable variability, with many outcomes either falling short or exceeding those of natural sites. The wide range of metrics used to assess restoration success further amplified the detection of this variability. Deviations from reference conditions did not decrease as restoration practices progressed over time, nor as a function of project spatial extent or age, highlighting persistent uncertainties. Restoration projects combining multiple interventions did not show improved alignment with reference conditions, nor reduced variability in outcomes, compared with single‐intervention approaches, suggesting that the expected benefits of combinatorial approaches may not be consistently realised across restoration contexts.

Despite the large variability, tidal restoration, sediment addition and fertilisation reproduced, on average, reference attributes, proving their value as tools for salt marsh restoration. Planting also restored, on average, physical and functional attributes, but consistently underperformed in species composition, such as cover, density, biomass or diversity of target and non‐target species. This limitation may be partially explained by findings from a recent meta‐analysis on planting success (Liu et al. 2021), which reported average survival rates of only 53% for plantings, including small‐scale transplantation experiments. Furthermore, the benefits of planted vegetation on species richness and diversity often require long time frames to develop (Dawe et al. 2000), while most studies in our analysis had relatively short post‐restoration monitoring periods. Additionally, some plant‐focused approaches intentionally use species that are different from native communities, selecting those more resistant to environmental change or more effective at providing desired ecosystem functions (van Hulzen et al. 2007). The trade‐offs of these approaches can be significant. For instance, De Battisti et al. (2024) have shown that monocultures like *Calamagrostis arenaria* can stabilise sand dunes but also limit the establishment of less competitive species, potentially reducing the overall biodiversity. These findings underscore that plant‐focused restoration should thoughtfully select species to support natural community development and ensure adequate long‐term monitoring (Brown and Amacher 1999; Palmer et al. 1997; Temmink et al. 2020).

The substantial variability observed in restoration outcomes aligns with patterns reported in terrestrial restoration meta‐analyses (Atkinson et al. 2022), as well as in other coastal systems (Sievers et al. 2024), suggesting some generalities in ecosystem responses to current restoration practices. In those studies and in ours, this variability was only partially explained by the type of intervention, spatial extent or age, suggesting that restoration success is strongly influenced by a range of unmeasured ecological and contextual factors, such as local environmental conditions, site‐specific implementation, and inherent differences among restored sites (Fraschetti et al. 2021; van der Heide et al. 2021). Such variability complicates the interpretation of restoration success, as outcomes are shaped not only by the type of interventions but also by the complex interactions of environmental and biological factors unique to each project (Airoldi et al. 2025).

While broader metrics are needed to gain a holistic understanding of ecosystem recovery (Fraschetti et al. 2021), the diversity of assessment metrics also adds complexity, hindering unequivocal evaluation and potentially affecting policymakers and stakeholders' perception of restoration value. To enhance consistency and impact, future research should aim to identify key drivers of this variability and develop standardised or better tailored evaluation frameworks across contexts.

It is also important to recognise that variability itself is not necessarily undesirable. While our analysis quantifies deviation from reference conditions, strict alignment with historical or “natural” baselines may not be feasible or optimal under accelerating climate change (Frietsch et al. 2023; Hobbs et al. 2009). Greater variability in structure and function can increase resilience, facilitate adaptation to shifting baselines, and support novel assemblages that continue to deliver key ecosystem function (Mayer‐Pinto et al. 2023; Suding 2011). From this perspective, some deviations from reference conditions may represent adaptive trajectories rather than failures (Cinner and Barnes 2019; Dudney et al. 2024), and metrics centred solely on reference similarity may overlook the value of functionally robust, though compositionally distinct, marsh communities. Moreover, the growing recognition that ecosystems are undergoing rapid, directional change challenges the assumption that historical reference conditions should always serve as the primary benchmark for success. Under climate‐driven shifts in temperature, hydrology, sediment regimes and species distributions, restored systems may follow novel but stable and functionally valuable trajectories. In such cases, variability in structure and function may represent adaptive reorganisation rather than deviation or failure. Embracing a broader envelope of acceptable outcomes (particularly those that sustain key functions such as coastal protection, carbon storage, or habitat provisioning) will be increasingly important for future‐proofing restoration under accelerating environmental change.

We found no evidence that spatial extent, timing, or age systematically explained restoration outcomes in the available dataset, suggesting that restoration performance may not be determined by scale or project age alone. Restoration outcomes did not appear to improve consistently with increasing project size or age, indicating that factors other than scale, such as site‐specific environmental conditions and implementation context, may play a stronger role in shaping restoration trajectories. This highlights the importance of locally tailored restoration approaches (Airoldi et al. 2025; Fraschetti et al. 2021; Meli et al. 2014).

Although salt marsh recovery can require decades, most published restoration studies focus on the first years following intervention, resulting in a strong bias towards younger age classes and limited representation of older restorations across intervention types. By modelling age as a continuous moderator, we explicitly accounted for recovery trajectories without assuming direct comparability between young, restored marshes and natural reference systems. The absence of a consistent age effect across physical, biotic and functional attributes suggests that the substantial residual variability among studies is not primarily driven by unaccounted recovery time but instead reflects strong context‐dependence and site‐specific ecological constraints.

Latitude was not a significant determinant of restoration success across most types of intervention, indicating that salt marsh restoration can be broadly applied across geographical regions with similar expectations for success. The exception was tidal restoration, where higher‐latitude sites showed lower success in replicating natural conditions. This may indicate the need to adapt tidal restoration techniques for colder or more temperate regions, as variations in hydrology or species' climate tolerance could affect salt marsh recovery (Wang et al. 2021; Zhang et al. 2019). Most studies included in this meta‐analysis were conducted in the Northern Hemisphere, particularly in North America and Europe, highlighting progress in these regions, but also underscoring the opportunity to expand ecological restoration efforts, research and investments in underrepresented regions, such as Africa and South America. Additionally, the focus on peer‐reviewed literature in English provides a robust foundation but may partly explain the geographic bias. Future meta‐analyses could integrate grey literature and studies published in other languages to gain a more comprehensive global perspective (Leo et al. 2019; Paez 2017).

Contrary to our original hypothesis, combinatorial interventions did not provide advantages over single targeted ones, neither in terms of alignment with reference conditions nor in the consistency of outcomes. While early combinatorial interventions showed effect sizes close to zero (indicating alignment with reference conditions), their success declined over time, diverging in both physical conditions and species composition. Additionally, outcome variability was also the highest recorded, while single interventions, such as tidal restoration, sediment addition, and fertilisation, consistently maintained an average match to reference states. These findings challenge the growing expectation that combining multiple restoration techniques will improve restoration outcomes (Rillig et al. 2024). However, these results do not necessarily indicate that combining restoration actions is inherently less effective than using single actions but instead reflect heterogeneity among combined interventions rather than a general effect. From an ecological perspective, this pattern is consistent with the idea that ecological processes are often sequential and context dependent. If combinatorial interventions are applied simultaneously or out of order, key stages (e.g., soil development, hydrology, colonisation) may be disrupted or could lead to unintended antagonistic interactions that hinder recovery. For example, a recent experiment showed that transplanted salt marsh vegetation had higher survival rates in undisturbed sediments than in those recently altered by sediment manipulation (De Lauretis 2025). This suggests that carrying out sediment addition and planting within the same restoration event could be counterproductive. Freshly placed sediment often requires time to consolidate, reduce physical instability, and develop suitable surface and subsurface conditions before it can support plant establishment and growth. If transplantation occurs too soon after sediment addition, plants may experience higher stress and lower survival rates due to unstable substrates and ongoing sediment settling. Overengineering could also interfere with natural recovery processes. For example, Warren et al. (2002) have explained how reestablishing tidal hydrology is often sufficient to initiate vegetation colonisation, and that planting may be unnecessary or even detrimental if done before hydrologic or sediment regimes stabilise. Other possible explanations for the lower performance of combinatorial approaches could include the increased operational complexity they introduce along with resource dilution, where limited funding or capacity might prevent each from being implemented successfully. Misaligned objectives between interventions (such as prioritising structural stability at the expense of biodiversity) could also create trade‐offs that reduce overall success. While the intention behind combinatorial approaches is to accelerate or reinforce restoration outcomes, the ecological reality is that multiple restoration interventions may interact in unpredictable ways, sometimes leading to antagonism. These interactions (whether through disrupted successional dynamics, operational challenges, or trade‐offs among restoration goals) underscore the need to better understand the conditions under which combined interventions foster positive synergies, if we aim to reduce current uncertainties.

As with any meta‐analysis, our findings must be interpreted in the context of structural limitations in the available literature. Restoration science is well known to suffer from systematic reporting, design, and publication biases. Successful, highly visible, or statistically significant outcomes are far more likely to be published than neutral, partial, or negative results, which are often relegated to grey literature or not reported at all (Earp et al. 2022). In addition, many studies lack explicit reporting of critical contextual variables (e.g., site conditions before intervention, implementation details, maintenance actions, or failures), which constrains the ability to identify mechanistic drivers of variability. Short monitoring periods further bias our understanding towards early post‐restoration trajectories, potentially missing later divergences, time lags, or delayed functional recovery. The geographic skew of available studies (heavily weighted towards North America and Europe) also limits global inference and likely reflects underlying disparities in research investment, monitoring capacity, and publishing accessibility. Taken together, these gaps mean that our quantitative synthesis necessarily reflects the structure of the accessible literature rather than the full spectrum of restoration outcomes worldwide.

The very notion of restoration ‘success’ requires careful consideration, as there is ongoing debate over how it should be defined and a persistent disconnect between how success is conceptualised in academic literature versus practice (Suding 2011; Wortley et al. 2013; Zedler 2007). Practitioners and academic researchers often use different criteria to define success, with practitioners prioritising operational feasibility, regulatory compliance, cost‐effectiveness, and the delivery of specific ecosystem services (Galbraith et al. 2021), while academic assessments more commonly focus on similarity to reference states and biological attributes (Clewell and Rieger 2008). This discrepancy means that projects considered only partially successful from an ecological standpoint may be regarded as successful by managers if they reduce erosion risk, enhance carbon sequestration, or improve habitat availability. Bridging this gap requires co‐developed metrics that integrate ecological realism with the constraints and objectives of on‐the‐ground implementation. Such integration would help ensure that restoration assessments reflect both scientific understanding and practitioner needs, ultimately improving the relevance and uptake of restoration science.

In conclusion, this study highlights both the potential and the limitations of current active salt marsh restoration practices. While most interventions approximated reference conditions on average, outcomes remained highly variable and were not systematically explained by project age, extent, or timing, underscoring the strong influence of site‐specific factors and the need for more context‐sensitive restoration strategies. Planting interventions require refinement, as their success depends not only on plant survival but also on fostering diverse and resilient communities over time. Importantly, combinatorial interventions did not outperform single, targeted approaches, challenging the assumptions that combining techniques inherently yields synergistic benefits. Rather, our findings point to substantial variability among combinatorial interventions. While combining interventions does not inherently reduce restoration success, more complex combinations may contribute to greater variability and less predictable outcomes.

Several strategies could strengthen current restoration practice and support ambitious global restoration goals. Future efforts should invest in identifying the most appropriate species and ensuring adequate post‐restoration monitoring to better align outcomes with natural community dynamics. A better understanding of how different interventions interact, when they create synergies versus when they hinder recovery, is essential, supported by experimental studies across ecological contexts and long‐term monitoring to capture delayed effects. Improved reporting standards and transparent documentation of objectives, implementation protocols, and local environmental conditions will enhance the comparability of studies and strengthen the evidence base for practice. Finally, decision‐support tools that integrate ecological insight with practical constraints (e.g., budget, site accessibility, climate resilience) can guide practitioners in tailoring strategies to local conditions and in recognising when combinatorial approaches are truly warranted.

## Author Contributions

**Laura Airoldi:** funding acquisition, conceptualization, methodology, supervision, project administration, writing – review and editing, writing – original draft. **Davide De Battisti:** conceptualization, methodology, writing – review and editing, supervision. **Serena De Lauretis:** conceptualization, methodology, investigation, validation, data curation, formal analysis, visualization, writing – original draft. **Ferrante Grasselli:** conceptualization, methodology, data curation, formal analysis, supervision.

## Funding

This work was supported by Italian Ministry of University and Research (MUR), DM 1062/2021, CN00000033.

## Conflicts of Interest

The authors declare no conflicts of interest.

## Supporting information


**Figure S1:** PRISMA diagram showing the flow of information applied in this review, from the literature search to the meta‐analyses.
**Figure S2:** Ven diagram showing the types of interventions and the relative proportions of combinatorial interventions included in the meta‐analysis.
**Figure S3:** Regression lines produced with meta‐regressions across all 492 effect sizes, illustrating the relationships between restoration success and Age for all restoration interventions. Dashed lines and dotted lines represent 95% confidence and 95% prediction interval, respectively. Individual data points are scaled by precision (1/SE) and colour‐coded by type of. Intervention. A statistically significant effect of Age was observed only for combinatorial approaches (*p* < 0.001).
**Figure S4:** Regression lines produced with meta‐regressions across all effect sizes (492) for each type of restoration intervention in interaction with Project timing (year). Dashed lines and dotted lines represent 95% confidence and 95% prediction interval respectively. Individual data points are scaled by precision (1/SE) and coloured by type of intervention.
**Figure S5:** Regression lines produced with meta‐regressions across all effect sizes (492) for each type of restoration interventions in interaction with Latitude. Dashed lines and dotted lines represent 95% confidence and 95% prediction interval respectively. Individual data points are scaled by precision (1/SE) and coloured by type of intervention. A statistically significant effect of Latitude was observed only for Tidal restoration (*p* < 0.001).
**Figure S6:** Leave‐one‐out sensitivity analysis of the meta‐analytic models across all combinatorial restoration approaches. The forest plot shows the pooled effect size estimates expressed as LnRR with 95% CI obtained after sequential exclusion of each category of combinatorial intervention from the full meta‐analytic model. Each leave‐one‐out estimate is compared against the full model estimate, which included all available observations (*k* = 203). The excluded intervention categories were sediment addition_plant manipulation (*k* = 58), sediment addition_plant manipulation_reef creation/fencing (*k* = 106), tidal restoration_sediment addition (*k* = 25) and sediment addition_reef creation/fencing (*k* = 25). For each sensitivity model, the number of observations represented in the forest plot corresponds to the total number of data points in the full model minus the number of observations associated with the excluded intervention category.
**Figure S7:** Leave‐one‐out sensitivity analysis of the meta‐analytic models across all combinatorial restoration approaches. The forest plot shows the pooled effect size estimates expressed as LnCVR with 95% CI obtained after sequential exclusion of each category of combinatorial intervention from the full meta‐analytic model. Each leave‐one‐out estimate is compared against the full model estimate, which included all available observations (*k* = 203). The excluded intervention categories were sediment addition_plant manipulation (*k* = 58), sediment addition_plant manipulation_reef creation/fencing (*k* = 106), tidal restoration_sediment addition (*k* = 25) and sediment addition_reef creation/fencing (*k* = 25). For each sensitivity model, the number of observations represented in the forest plot corresponds to the total number of data points in the full model minus the number of observations associated with the excluded intervention category.
**Table S1:** Studies included in the meta‐analysis (*n* = 34), with indication of their key metadata. All studies formally compared restored and natural (reference) salt marshes.
**Table S2:** Results of the meta‐analysis of the effects of different types of restoration interventions (Tidal restoration, Sediment addition, Planting, Fertilisation and Combinatorial) and outcome attributes: Ecosystem function, Physical conditions, and Species composition. The model results section reports the estimates effects of each interaction between restoration intervention and outcome attributes, including standard errors (SE), *z*‐values, *p*‐values and 95% confidence intervals (CI).
**Table S3:** Results of the meta‐analysis of the effects of different types of restoration interventions (Tidal restoration, Sediment addition, Planting, Fertilisation and Combinatorial) and Age of the intervention. The model results section displays the estimates for each interaction between intervention and Age, including standard errors (SE), *z*‐values, *p*‐values and 95% confidence intervals (CI).
**Table S4:** Results of the meta‐analysis model that evaluated the interactions between different types of restoration interventions (Tidal restoration, Sediment addition, Planting, Fertilisation and Combinatorial) and Project timing (year) of the interventions including standard errors (SE), *z*‐values, *p*‐values and 95% confidence intervals (CI).
**Table S5:** Results of the meta‐analysis model that evaluated the interactions between different types of restoration interventions (Tidal restoration, Sediment addition, Planting, Fertilisation and Combinatorial) and Spatial extent of the intervention. The model results section displays the estimates for each interaction between intervention and Spatial extent, including standard errors (SE), *z*‐values, *p*‐values and 95% confidence intervals (CI).
**Table S6:** Results of the meta‐analysis model that evaluated the interactions between different types of restoration interventions (Tidal restoration, Sediment addition, Planting, Fertilisation and Combinatorial) and Latitude. The model results section displays the estimates for each interaction between Intervention and Latitude, including standard errors (SE), *z*‐values, *p*‐values and 95% confidence intervals (CI).
**Table S7a:** Results of the meta‐analysis of the effects of different types of restoration interventions (Tidal restoration, Sediment addition, Planting, Fertilisation and Combinatorial). The model results section displays the estimates of effect size (lnRR) for each intervention, including standard errors (SE), *z*‐values, *p*‐values and 95% confidence intervals (CI).
**Table S7b:** Results of the meta‐analysis of the effects of different types of restoration interventions (Tidal restoration, Sediment addition, Planting, Fertilisation and Combinatorial). The model results section displays the estimates of coefficient of variation (lnCVR) for each intervention, including standard errors (SE), *z*‐values, *p*‐values and 95% confidence intervals (CI).
**Table S8:** Results of the meta‐analysis model that evaluated the interaction between different types of restoration interventions (Tidal restoration, Sediment addition, Planting, Fertilisation and Combinatorial) and Age of the intervention. It presents the QE (residual heterogeneity statistic), degrees of freedom (df), and *p*‐values for both QE and QM (moderator test statistic), indicating the statistical significance of the findings.
**Table S9:** Results of the meta‐analysis model that evaluated the interactions between different types of restoration interventions (Tidal restoration, Sediment addition, Planting, Fertilisation and Combinatorial) and Project timing (years) of the intervention. It presents the QE (residual heterogeneity statistic), degrees of freedom (df), and *p*‐values for both QE and QM (moderator test statistic), indicating the statistical significance of the findings.
**Table S10:** Results of the meta‐analysis model that evaluated the interactions between different types of restoration interventions (Tidal restoration, Sediment addition, Planting, Fertilisation and Combinatorial) and Spatial extent of the intervention. It presents the QE (residual heterogeneity statistic), degrees of freedom (df), and *p*‐values for both QE and QM (moderator test statistic), indicating the statistical significance of the findings.
**Table S11:** Results of the meta‐analysis model that evaluated the interactions between different types of restoration interventions (Tidal restoration, Sediment addition, Planting, Fertilisation and Combinatorial/) and Latitude of the intervention. It presents the QE (residual heterogeneity statistic), degrees of freedom (df), and *p*‐values for both QE and QM (moderator test statistic), indicating the statistical significance of the findings.
**Table S12:** Leave‐one‐out sensitivity analysis for combinatorial interventions. The model results section displays the estimates of effect size (lnRR) for each model with the excluded intervention, including standard errors (SE), 95% confidence intervals (CI) and *p*‐values.
**Table S13:** Leave‐one‐out sensitivity analysis for combinatorial interventions. The model results section displays the coefficient of variation (lnCVR) for each model with the excluded intervention, including standard errors (SE), 95% confidence intervals (CI) and *p*‐values.

## Data Availability

The data that support the findings of this study are openly available in Zenodo at: https://doi.org/10.5281/zenodo.20071735.
