# Supplementary material for: A Global Review of Salt Marsh Restoration: Why Simpler, Context‐Specific Strategies Can Outperform Complex Approaches
Source: Glob Chang Biol. 2026 May 21;32(5):e70925. doi: 10.1111/gcb.70925 (PMC13602432; doi:10.1111/gcb.70925)
Supplement: Supplementary file 1 [file GCB-32-e70925-s001.docx]

**SUPPLEMENTARY INFORMATION**

***Figure S1****. PRISMA diagram showing the flow of information applied in this review, from the literature search to the meta-analyses.*

***Table S1****: Studies included in the meta-analysis (n=34), with indication of their key metadata. All studies formally compared restored and natural (reference) salt marshes.*

| Authors | Study_ID | Exp_ID | Year | Continent | Type of manipulation |  | Extension (ha) | Age (years) |
| --- | --- | --- | --- | --- | --- | --- | --- | --- |
| Abbot et al. | 40 | 26 | 2019 | North America | Single manipulation | Sediment addition | 71-115 | 8-20 |
| Able et al. | 54 | 41 | 2008 | North America | Combinatorial | Combinatorial | 148 | 3-4 |
| Armitage et al. | 4 | 4 | 2006 | North America | Single manipulation | Planting | 0.03 | 3 |
| Baas et al. | 23 | 18 | 2014 | North America | Single manipulation | Planting | 0.07 | 3 |
| Baustian et al. | 34 | 19 | 2006 | North America | Single manipulation | Sediment addition | 4.7 | 20 |
| Boyer et al. | 6 | 8 | 1998 | North America | Single manipulation | Fertilisation | 0.03 | 0.5 |
| Broome et al. | 61 | 44 | 1982 | North America | Single manipulation | Planting | 44 | 2-3 |
| Burdick et al. | 49 | 34 | 1996 | North America | Single manipulation | Tidal restoration | 4.5-40 | 2-7.5 |
| Cai et al. | 17 | 14 | 2022 | Europe | Single manipulation | Tidal restoration | 22 | 2 |
| Craft et al. | 62 | 46 | 2003 | North America | Single manipulation | Planting | 0.5-1 | 5-25 |
| Darby et al. | 36 | 21 | 2008 | North America | Single manipulation | Fertilisation | 0.003 | 0.5 |
| Davis et al. | 63 | 47 | 2017 | North America | Single manipulation | Fertilisation | 0.01 | 3 |
| Davis et al. | 64 | 48 | 2015 | North America | Combinatorial | Combinatorial | 0.05 | 11 |
| Davis et al. | 64 | 49 | 2015 | North America | Single manipulation | Planting | 0.05 | 1 |
| Edwards et al. | 37 | 22 | 2003 | North America | Single manipulation | Sediment addition | 40-200 | 3-19 |
| Ford et al. | 38 | 23 | 1999 | North America | Single manipulation | Sediment addition | 0.5 | 1-2 |
| Gallego et al. | 71 | 52 | 2007 | Europe | Combinatorial | Combinatorial | 52 | 5 |
| Huspeni et al. | 10 | 9 | 2004 | North America | Combinatorial | Combinatorial | 6 | 6 |
| Isdell et al. | 80 | 56 | 2021 | North America | Combinatorial | Combinatorial | 0.5-1.2 | 2-16 |
| Kadiri et al. | 18 | 15 | 2011 | Europe | Combinatorial | Combinatorial | 108 | 2 |
| Lawrence et al. | 19 | 16 | 2022 | Europe | Combinatorial | Combinatorial | 66 | 14 |
| Madrid et al. | 75 | 53 | 2012 | North America | Combinatorial | Combinatorial | 45 | 2.5 |
| McAtee et al. | 12 | 11 | 2020 | North America | Single manipulation | Sediment addition | 4.05 | 0.5 |
| McClellan et al. | 40 | 25 | 2021 | North America | Single manipulation | Sediment addition | 7.5-80 | 1-33 |
| Minello et al. | 92 | 60 | 1997 | North America | Single manipulation | Planting | 1 | 6 |
| Moseman-Valtierra et al. | 94 | 63 | 2016 | Europe | Combinatorial | Combinatorial | 12.5 | 10-14 |
| Nordstrom et al. | 94 | 64 | 2016 | Europe | Combinatorial | Combinatorial | 12.5 | 8 |
| Perry et al. | 69 | 50 | 2022 | North America | Single manipulation | Sediment addition | Not specified | 5 |
| Puchkoff et al. | 16 | 13 | 2022 | North America | Single manipulation | Sediment addition | 0.4 | 1 |
| Rozas et al. | 44 | 29 | 2001 | North America | Combinatorial | Combinatorial | 10 | 9 |
| Rozas et al. | 77 | 54 | 2007 | North America | Combinatorial | Combinatorial | 1.7 | 3 |
| Stagg et al. | 45 | 30 | 2010 | North America | Single manipulation | Sediment addition | 7.5 | 2 |
| Staszak et al. | 78 | 55 | 2013 | North America | Single manipulation | Planting | 0.3 | 5-15 |
| Tong et al. | 45 | 31 | 2013 | North America | Single manipulation | Sediment addition | 7.5 | 4 |
| Tong et al. | 45 | 32 | 2013 | North America | Single manipulation | Sediment addition | 7.5 | 7 |
| Van Proosdij et al. | 15 | 12 | 2010 | North America | Single manipulation | Tidal restoration | 10 | 3 |
| VanZomeren et al. | 56 | 43 | 2018 | North America | Single manipulation | Sediment addition | 44 | 0.5 |
| Wang et al. | 50 | 36 | 2021 | North America | Combinatorial | Combinatorial | 15 | 11 |
| Wang et al. | 50 | 37 | 2021 | North America | Combinatorial | Combinatorial | 15 | 10 |
| Wang et al. | 50 | 38 | 2021 | North America | Combinatorial | Combinatorial | 15 | 8 |
| Wang et al. | 50 | 39 | 2021 | North America | Combinatorial | Combinatorial | 15 | 6 |

***Figure S2*** *Ven diagram showing the types of interventions and the relative proportions of combinatorial interventions included in the meta-analysis*

***Table S2*** *Results of the meta-analysis of the effects of different types of restoration interventions (Tidal restoration, Sediment addition, Planting, Fertilisation and Combinatorial) and outcome attributes: Ecosystem function, Physical conditions, and Species composition. The model results section reports the estimates effects of each interaction between restoration intervention and outcome attributes, including standard errors (SE), z-values, p-values, and 95% confidence intervals (CI).*

| **Attribute & Intervention** | **Estimate (lnRR)** | **SE** | **z-value** | **p-value** | **95% CI (Lower)** | **95% CI (Upper)** | **Significance^1^** |
| --- | --- | --- | --- | --- | --- | --- | --- |
| Ecosystem function: Fertilisation | 0.3328 | 0.2809 | 1.1846 | 0.2362 | -0.2178 | 0.8833 |  |
| Physical conditions: Fertilisation | 0.1431 | 0.2922 | 0.4898 | 0.6243 | -0.4295 | 0.7157 |  |
| Ecosystem function: Combinatorial | -0.1052 | 0.1217 | -0.8643 | 0.3874 | -0.3436 | 0.1333 |  |
| Physical conditions: Combinatorial | -0.3999 | 0.1239 | -3.2268 | 0.0013 | -0.6428 | -0.157 | ** |
| Species composition: Combinatorial | -0.4189 | 0.1253 | -3.3419 | 0.0008 | -0.6645 | -0.1732 | *** |
| Ecosystem function: Planting | 0.1982 | 0.1317 | 1.5052 | 0.1323 | -0.0599 | 0.4562 |  |
| Physical conditions: Planting | 0.1936 | 0.1392 | 1.3908 | 0.1643 | -0.0792 | 0.4664 |  |
| Species composition: Planting | -1.06 | 0.2049 | -5.1741 | <.0001 | -1.4615 | -0.6584 | *** |
| Ecosystem function: Sediment addition | -0.2718 | 0.1552 | -1.751 | 0.0799 | -0.576 | 0.0324 |  |
| Physical conditions: Sediment addition | 0.0762 | 0.1536 | 0.4963 | 0.6197 | -0.2248 | 0.3773 |  |
| Species composition: Sediment addition | 0.0334 | 0.1575 | 0.2119 | 0.8322 | -0.2753 | 0.342 |  |
| Ecosystem function: Tidal restoration | -0.0182 | 0.2264 | -0.0803 | 0.936 | -0.462 | 0.4256 |  |
| Physical conditions: Tidal restoration | -0.0626 | 0.2227 | -0.2811 | 0.7786 | -0.4991 | 0.3739 |  |
| Species composition: Tidal restoration | 0.4035 | 0.2485 | 1.6239 | 0.1044 | -0.0835 | 0.8905 |  |
| *^1^** p < 0.05; ** p < 0.01; *** p < 0.001 | | | | | | | |

***Table S3*** *Results of the meta-analysis of the effects of different types of restoration interventions (Tidal restoration, Sediment addition, Planting, Fertilisation and Combinatorial) and age of the intervention. The model results section displays the estimates for each interaction between intervention and age, including standard errors (SE), z-values, p-values, and 95% confidence intervals (CI).*

| **Variable** | **Estimate (lnRR)** | **SE** | **Z-value** | **P-value** | **95% CI (Lower)** | **95% CI (Upper)** | **Significance^1^** |
| --- | --- | --- | --- | --- | --- | --- | --- |
| Age | 0.0196 | 0.0410 | 0.4784 | 0.6324 | -0.0607 | 0.0999 |  |
| Fertilisation | 0.2651 | 0.3375 | 0.7856 | 0.4321 | -0.3964 | 0.9267 |  |
| Combinatorial | -0.9421 | 0.1509 | -6.2419 | <.0001 | -1.2380 | -0.6463 | *** |
| Planting | 0.1825 | 0.1517 | 1.2024 | 0.2292 | -0.1150 | 0.4799 |  |
| Sediment addition | 0.0923 | 0.1847 | 0.4996 | 0.6173 | -0.2697 | 0.4543 |  |
| Tidal restoration | -0.0066 | 0.3445 | -0.0191 | 0.9847 | -0.6819 | 0.6687 |  |
| Age: Combinatorial | 0.0606 | 0.0414 | 1.4626 | 0.1436 | -0.0206 | 0.1418 |  |
| Age: Planting | -0.0023 | 0.0410 | -0.0552 | 0.956 | -0.0826 | 0.0781 |  |
| Age: Sediment addition | -0.0261 | 0.0410 | -0.6361 | 0.5247 | -0.1064 | 0.0543 |  |
| Age: Tidal restoration | -0.0326 | 0.0611 | -0.5342 | 0.5932 | -0.1524 | 0.0871 |  |

*^1^** p < 0.05; ** p < 0.01; *** p < 0.001

***Figure S3*** *Regression lines produced with meta-regressions across all 492 effect sizes, illustrating the relationships between restoration success and Age for all restoration interventions. Dashed lines and dotted lines represent 95% confidence and 95% prediction interval, respectively. Individual data points are scaled by precision (1/SE) and color-coded by type of. Intervention. A statistically significant effect of age was observed only for combinatorial approaches (p<0.001).*

***Table S4****: Results of the meta-analysis model that evaluated the interactions between different types of restoration interventions (Tidal restoration, Sediment addition, Planting, Fertilisation and Combinatorial) and project timing (year) of the interventions including standard errors (SE), z-values, p-values, and 95% confidence intervals (CI).*

| **Variable** | **Estimate (lnRR)** | **Standard Error** | **Z-Value** | **P-value** | **95% CI (Lower)** | **95% CI (Upper)** | **Significance^1^** |
| --- | --- | --- | --- | --- | --- | --- | --- |
| Project timing (Year) | 0.0159 | 0.0259 | 0.6124 | 0.5403 | -0.0349 | 0.0666 |  |
| Fertilisation | -31.4581 | 51.8517 | -0.6067 | 0.5441 | -133.0857 | 70.1694 |  |
| Combinatorial | 158.5878 | 12.3444 | 12.8470 | <.0001 | 134.3933 | 182.7823 | *** |
| Planting | -30.0940 | 3.5971 | -8.3662 | <.0001 | -37.1441 | -23.0438 | *** |
| Sediment addition | -12.2707 | 3.5185 | -3.4875 | 5e-04 | -19.1667 | -5.3746 | *** |
| Tidal restoration | 20.3385 | 44.1327 | 0.4608 | 0.6449 | -66.1601 | 106.8371 |  |
| Year: Combinatorial | -0.0953 | 0.0266 | -3.5787 | 3e-04 | -0.1474 | -0.0431 | *** |
| Year: Planting | -0.0008 | 0.0260 | -0.0292 | 0.9767 | -0.0516 | 0.0501 |  |
| Year: Sediment addition | -0.0097 | 0.0260 | -0.3742 | 0.7083 | -0.0606 | 0.0412 |  |
| Year: Tidal restoration | -0.0260 | 0.0340 | -0.7660 | 0.4437 | -0.0927 | 0.0406 |  |

*^1^** p < 0.05; ** p < 0.01; *** p < 0.001

***Figure S4****. Regression lines produced with meta-regressions across all effect sizes (492) for each type of restoration intervention in interaction with Project timing (year). Dashed lines and dotted lines represent 95% confidence and 95% prediction interval respectively. Individual data points are scaled by precision (1/SE) and coloured by type of intervention.*

***Table S5****: Results of the meta-analysis model that evaluated the interactions between different types of restoration interventions (Tidal restoration, Sediment addition, Planting, Fertilisation and Combinatorial) and spacial extent of the intervention. The model results section displays the estimates for each interaction between intervention and age, including standard errors (SE), z-values, p-values, and 95% confidence intervals (CI).*

| **Variable** | **Estimate (lnRR)** | **SE** | **Z-Value** | **P-value** | **95% CI (Lower)** | **95% CI (Upper)** | **Significance^1^** |
| --- | --- | --- | --- | --- | --- | --- | --- |
| Spacial extent | 13.4581 | 24.7042 | 0.5448 | 0.5859 | -34.9613 | 61.8776 |  |
| Fertilisation | 0.0957 | 0.4632 | 0.2066 | 0.8363 | -0.8121 | 1.0035 |  |
| Combinatorial | -0.2220 | 0.1552 | -1.4306 | 0.1526 | -0.5263 | 0.0822 |  |
| Planting | 0.1242 | 0.1584 | 0.7845 | 0.4328 | -0.1862 | 0.4346 |  |
| Sediment addition | 0.0322 | 0.1681 | 0.1916 | 0.8481 | -0.2973 | 0.3618 |  |
| Tidal restoration | -0.0035 | 0.2880 | -0.0123 | 0.9902 | -0.5680 | 0.5609 |  |
| Extension: Combinatorial | -13.4566 | 24.7042 | -0.5447 | 0.5860 | -61.8760 | 34.9628 |  |
| Extension: Planting | -13.4874 | 24.7042 | -0.5460 | 0.5851 | -61.9069 | 34.9320 |  |
| Extension: Sediment addition | -13.4576 | 24.7042 | -0.5448 | 0.5859 | -61.8771 | 34.9618 |  |
| Extension: Tidal restoration | -13.4618 | 24.7042 | -0.5449 | 0.5858 | -61.8812 | 34.9577 |  |

*^1^** p < 0.05; ** p < 0.01; *** p < 0.001

***Table S6****: Results of the meta-analysis model that evaluated the interactions between different types of restoration interventions (Tidal restoration, Sediment addition, Planting, Fertilisation and Combinatorial) and Latitude. The model results section displays the estimates for each interaction between Intervention and Latitude, including standard errors (SE), z-values, p-values, and 95% confidence intervals (CI).*

| **Variable** | **Estimate (lnRR)** | **SE** | **Z-value** | **P-value** | **95% CI (Lower)** | | **95% CI (Upper)** | **Significance^1^** |
| --- | --- | --- | --- | --- | --- | --- | --- | --- |
| Latitude | 0.1823 | 0.1009 | 1.8061 | 0.0709 | | -0.0155 | 0.3802 |  |
| Fertilisation | -5.8516 | 3.4119 | -1.7150 | 0.0863 | | -12.5388 | 0.8357 |  |
| Combinatorial | -0.6851 | 0.6837 | -1.0021 | 0.3163 | | -2.0251 | 0.6548 |  |
| Planting | 1.9571 | 2.5588 | 0.7649 | 0.4444 | | -3.0580 | 6.9722 |  |
| Sediment addition | -0.8266 | 1.0276 | -0.8044 | 0.4212 | | -2.8405 | 1.1874 |  |
| Tidal restoration | 2.4878 | 2.3147 | 1.0748 | 0.2825 | | -2.0489 | 7.0244 |  |
| Latitude: Combinatorial | -0.1718 | 0.1025 | -1.6763 | 0.0937 | | -0.3726 | 0.0291 |  |
| Latitude: Planting | -0.2393 | 0.1257 | -1.9040 | 0.0569 | | -0.4856 | 0.0070 |  |
| Latitude: Sediment addition | -0.1568 | 0.1053 | -1.4896 | 0.1363 | | -0.3631 | 0.0495 |  |
| Latitude: Tidal restoration | -0.2362 | 0.1120 | -2.1080 | 0.0350 | | -0.4558 | -0.0166 | * |

*^1^** p < 0.05; ** p < 0.01; *** p < 0.001

***Figure S5****. Regression lines produced with meta-regressions across all effect sizes (492) for each type of restoration interventions in interaction with Latitude. Dashed lines and dotted lines represent 95% confidence and 95% prediction interval respectively. Individual data points are scaled by precision (1/SE) and coloured by type of intervention. A statistically significant effect of age was observed only for Tidal restoration (p<0.001).*

***Table S7a*** *Results of the meta-analysis of the effects of different types of restoration interventions (Tidal restoration, Sediment addition, Planting, Fertilisation and Combinatorial. The model results section displays the estimates of effect size (lnRR) for each intervention, including standard errors (SE), z-values, p-values, and 95% confidence intervals (CI).*

| **Moderator** | **Estimate (lnRR)** | **SE** | **z-value** | **p-value** | **95% CI (Lower)** | **95% CI (Upper)** | **Significance^1^** |
| --- | --- | --- | --- | --- | --- | --- | --- |
| Combinatorial | -0.2548 | 0.1174 | -2.1711 | 0.0299 | -0.4848 | -0.0248 | * |
| Sediment addition | 0.0444 | 0.1491 | 0.2978 | 0.7658 | -0.2478 | 0.3366 |  |
| Fertilisation | 0.2923 | 0.2707 | 1.0796 | 0.2803 | -0.2384 | 0.8229 |  |
| Planting | 0.0352 | 0.1275 | 0.2758 | 0.7827 | -0.2148 | 0.2852 |  |
| Tidal restoration | -0.0669 | 0.2175 | -0.3073 | 0.7586 | -0.4932 | 0.3595 |  |

*^1^** p < 0.05; ** p < 0.01; *** p < 0.001

***Table S7b*** *Results of the meta-analysis of the effects of different types of restoration interventions (Tidal restoration, Sediment addition, Planting, Fertilisation and Combinatorial. The model results section displays the estimates of coefficient of variation (lnCVR) for each intervention, including standard errors (SE), z-values, p-values, and 95% confidence intervals (CI).*

| **Moderator** | **Estimate (lnCVR)** | **SE** | **z-value** | **p-value** | **95% CI (Lower)** | **95% CI (Upper)** | **Significance^1^** |
| --- | --- | --- | --- | --- | --- | --- | --- |
| Combinatorial | 1.0633 | 0.2461 | 4.3204 | <0.0001 | 0.5809 | 1.5457 | *** |
| Sediment addition | 0.5213 | 0.3131 | 1.6652 | 0.0959 | -0.0923 | 1.1350 |  |
| Fertilisation | 0.0278 | 0.5577 | 0.0499 | 0.9602 | -1.0653 | 1.1209 |  |
| Planting | -0.6769 | 0.2790 | -2.4264 | 0.0152 | -1.2236 | -0.1301 | * |
| Tidal restoration | 0.4759 | 0.4655 | 1.0224 | 0.3066 | -0.4364 | 1.3883 |  |

*^1^** p < 0.05; ** p < 0.01; *** p < 0.001

***Table S8*** *Summary outcomes of the meta-analysis of the effects of different types of restoration interventions (Tidal restoration, Sediment addition, Planting, Fertilisation and Combinatorial) and the age of the intervention. It presents the QE (residual heterogeneity statistic), degrees of freedom (df), and p-values for both QE and QM (moderator test statistic), indicating the statistical significance of the findings.*

| **Test** | **Statistic** | **df** | **Value** | **P-value** |
| --- | --- | --- | --- | --- |
| Residual Heterogeneity | QE | 482 | 11,012.5619 | < .0001 |
| Test of Moderators (coefficients 1:10) | QM | 10 | 284.0899 | < .0001 |

Note: p-val < .0001 indicates statistical significance at alpha = 0.001 level.

***Table S9****: Results of the meta-analysis model that evaluated the interactions between different types of restoration interventions (Tidal restoration, Sediment addition, Planting, Combinatorial, and Fertilisation) and project timing (years). It presents the QE (residual heterogeneity statistic), degrees of freedom (df), and p-values for both QE and QM (moderator test statistic), indicating the statistical significance of the findings.*

| **Test** | **Statistic** | **df** | **Value** | **P-value** |
| --- | --- | --- | --- | --- |
| Residual Heterogeneity | QE | 482 | 10,579.9200 | < .0001 |
| Test of Moderators (coefficients 1:10) | QM | 10 | 260.8739 | < .0001 |

Note: p-val < .0001 indicates statistical significance at alpha = 0.001 level.

***Table S10****: Results of the meta-analysis model that evaluated the interactions between different types of restoration interventions (Tidal restoration, Sediment addition, Planting, Fertilisation and Combinatorial) and intervention extension. It presents the QE (residual heterogeneity statistic), degrees of freedom (df), and p-values for both QE and QM (moderator test statistic), indicating the statistical significance of the findings.*

| **Test** | **Statistic** | **df** | **Value** | **P-value** |
| --- | --- | --- | --- | --- |
| Residual Heterogeneity | QE | 476 | 11,178.3763 | < .0001 |
| Test of Moderators (coefficients 1:10) | QM | 10 | 22.9740 | 0.0108 |

Note: p-val < .0001 indicates high statistical significance.

***Table S11****: Results of the meta-analysis model that evaluated the interactions between different types of restoration interventions (Tidal restoration, Sediment addition, Planting, Fertilisation and Combinatorial/Combinatorial) and Latitude. It presents the QE (residual heterogeneity statistic), degrees of freedom (df), and p-values for both QE and QM (moderator test statistic), indicating the statistical significance of the findings.*

| **Test** | **Statistic** | **df** | **Value** | **P-value** |
| --- | --- | --- | --- | --- |
| Residual Heterogeneity | QE | 482 | 10,705.6378 | < .0001 |
| Test of Moderators (coefficients 1:10) | QM | 10 | 18.5416 | 0.0465 |

Note: p-val < .0001 indicates statistical significance at alpha = 0.001 level.

***Table S12:*** *Leave-one-out sensitivity analysis for combinatorial interventions. The model results section displays the estimates of effect size (lnRR) for each model with the excluded intervention, including standard errors (SE), 95% confidence intervals (CI) and p-values.*

| **Scenario (excluded subtype)** | **Estimate (lnRR)** | **SE** | **95% CI** | **p-value** |
| --- | --- | --- | --- | --- |
| Full model (no exclusion) | 1.15 | 0.25 | [0.65, 1.65] | <0.0001 |
| Sediment addition + planting | -0.36 | 0.14 | [-0.63, -0.09] | 0.01 |
| Sediment addition + tidal restoration | -0.27 | 0.14 | [-0.55, 0.01] | 0.06 |
| **Sediment addition + planting + reef creation and fencing** | **-0.08** | **0.15** | **[-0.37, 0.22]** | **0.61** |
| Sediment addition + reef creation and fencing | -0.27 | 0.12 | [-0.51, -0.02] | 0.03 |

***Table S13:*** *Leave-one-out sensitivity analysis for combinatorial interventions. The model results section displays the coefficient of variation (lnCVR) for each model with the excluded intervention, including standard errors (SE), 95% confidence intervals (CI) and p-values.*

| **Scenario (excluded subtype)** | **Estimate (lnRR)** | **SE** | **95% CI** | **p-value** |
| --- | --- | --- | --- | --- |
| Full model (no exclusion) | 1.15 | 0.25 | [0.65, 1.65] | < 0.0001 |
| Sediment addition + planting | 1.27 | 0.29 | [0.69, 1.84] | < 0.0001 |
| Sediment addition + tidal restoration | 1.31 | 0.30 | [0.72, 1.90] | < 0.0001 |
| **Sediment addition + planting + reef creation and fencing** | 0.42 | 0.23 | [-0.02, 0.87] | 0.06 |
| Sediment addition + reef creation and fencing | 1.15 | 0.25 | [0.65, 1.65] | < 0.0001 |

***Figure S6.*** *Leave-one-out sensitivity analysis of the meta-analytic models across all combinatorial restoration approaches. The forest plot shows the pooled effect size estimates expressed as LnRR with 95% CI obtained after sequential exclusion of each category of combinatorial intervention from the full meta-analytic model. Each leave-one-out estimate is compared against the full model estimate, which included all available observations (k = 203). The excluded intervention categories were sediment addition_plant manipulation (k = 58), sediment addition_plant manipulation_reef creation/fencing (k = 106), tidal restoration_sediment addition (k = 25), and sediment addition_reef creation/fencing (k = 25). For each sensitivity model, the number of observations represented in the forest plot corresponds to the total number of data points in the full model minus the number of observations associated with the excluded intervention category.*

***Figure S7.*** *Leave-one-out sensitivity analysis of the meta-analytic models across all combinatorial restoration approaches. The forest plot shows the pooled effect size estimates expressed as LnCVR with 95% CI obtained after sequential exclusion of each category of combinatorial intervention from the full meta-analytic model. Each leave-one-out estimate is compared against the full model estimate, which included all available observations (k = 203). The excluded intervention categories were sediment addition_plant manipulation (k = 58), sediment addition_plant manipulation_reef creation/fencing (k = 106), tidal restoration_sediment addition (k = 25), and sediment addition_reef creation/fencing (k = 25). For each sensitivity model, the number of observations represented in the forest plot corresponds to the total number of data points in the full model minus the number of observations associated with the excluded intervention category.*
